# Novel bioparameters derived from bioimpedance measurements for accurate prediction of weight status in infant–juvenile individuals: A regression analysis

**DOI:** 10.2478/joeb-2025-0009

**Published:** 2025-05-26

**Authors:** Taira Batista Luna, Jose Luis García Bello, Alcibíades Lara Lafargue, Héctor Manuel Camué Ciria, Yohandys A. Zulueta

**Affiliations:** Autonomous University of Santo Domingo (UASD), UASD Nagua Center, Dominican Republic; Autonomous University of Santo Domingo (UASD), San Francisco de Macorís Campus, Dominican Republic; National Center for Applied Electromagnetism (CNEA), Universidad de Oriente CP 90500, Santiago de Cuba, Cuba; Departamento de Física, Facultad de Ciencias Naturales y Exactas, Universidad de Oriente, CP 90500, Santiago de Cuba, Cuba

**Keywords:** Bioimpedance, biomedical parameters, body mass index, machine learning, phase angle

## Abstract

In this study, a linear support vector machine regression model was used to explore the correlation between weight status and two novel bioparameters, specific resistance and reactance, in an infant-juvenile cohort from eastern Cuba. The model was trained using various characteristics, including bioimpedance measurements, to predict phase angle, specific resistance, and reactance with high accuracy. The results showed that the variation of these characteristics with weight status and sex is consistent with previous literature. Additionally, two robust bioparameters derived from bioimpedance measurements and anthropometric-physiological parameters were identified for predicting weight status. The predictive models developed in this study are essential for accurately assessing weight status and disease risks in infants and juveniles in the eastern Cuban region. These findings highlight the potential applications of bioimpedance measurements and bioparameters in health and disease risk assessment, contributing to the growing body of literature on this topic.

## Introduction

Bioimpedance is a non-invasive technique used to measure the electrical impedance or resistance of biological tissues or fluids by applying an electrical current through the skin and measuring the resulting voltage [1,2,3,4]. In addition, bioimpedance techniques provide valuable information on various physiological parameters, including body composition, tissue health, cell membrane integrity, hydration status, the measurement of extracellular and intracellular water (ECW and ICW, respectively), and total body water (TBW) [5,6,7,8,9].

Bioimpedance is particularly useful in detecting dehydration, overhydration, and excess fluid accumulation in renal patients treated by hemodialysis. The development of bioimpedance methods has emerged as an alternative non-invasive method to obtain access to these physiological parameters [1,2,3,4,5,6,7,8,9].

The health status of individuals is closely associated with their weight status [10,11,12,13,14,15,16,17,18]. Obesity is a significant risk factor for the development of type diabetes, hypertension, heart disease, and chronic kidney disease (CKD) [10,11,19]. The incidence of obesity on the health status has increased monotonically [12]. On the other hand, underweight is also a risk factor for general health status and has been linked to lung diseases, fractures, cardiovascular diseases, atrial fibrillation and chronic kidney disease [11,12,13,14,15,20,21].

The World Health Organization (WHO) specifically quantifies the proportion of fat mass relative to total body weight using the body mass index (BMI) and has established various classes for differentiating weight status [22]. However, according to WHO classification of weight status, demographic age distributions vary significantly across different countries and age groups. This variation can lead to incorrect diagnoses of weight status when comparing global regions and age groups [11,22]. Machine learning is a subfield of artificial intelligence that enables computers to learn from data and make predictions or decisions without being explicitly implemented as a computer code [23,24,25,26]. In the medicine field, machine learning has the potential of providing more precise diagnoses, helping in decision-making and personalized treatment plans [26,27,28].

In a previous work a combination of bioimpedance measurements and machine learning was performed in an infant-juvenile cohort form the eastern Cuban region [28]. The classification model demonstrated that there are other indicators instead of body mass index that can be used as a predictors of weight status (the size, body density, phase angle, body mass index, fat-free mass (FFMS), total body water volume according to Kotler, body surface area, extracellular water according to Kotler, and sex) [28]. In addition, the regression learner model predicted with high accuracy the weight status of the volunteers [28].

Although bioimpedance provides valuable physiological information, there is no association between bioelectrical parameters and weight status. Further studies are required to address these issues. The aim of this research paper is to develop and validate predictive models to explore the relationship between weight status and novel bioparameters, specifically specific resistance and reactance, in an infant-juvenile cohort from eastern Cuban region. By leveraging bioimpedance measurements and various anthropometric-physiological characteristics, the study aims to accurately predict phase angle, specific resistance, and reactance. The ultimate goal is to enhance the assessment of weight status and disease risks in young individuals, providing valuable insights into the potential applications of these bioparameters in health and disease risk evaluation. This research contributes to existing literature by identifying robust bioparameters for predicting weight status and highlighting their significance in health assessments.

## Materials and methods

As in a previous study [28], a pilot randomized trial was conducted at the Pediatrics Hospital in Santiago de Cuba. The study, which took place between 2002 and 2008, involved 283 male and female volunteers aged 2 to 18 years. Bioimpedance parameters were measured using a BioScan^®^ 98 (Biológica Tecnología Médica SL, Barcelona, España) analyzer and the standard tetrapolar whole-body configuration was used. Volunteers were required to fast for at least three hours and empty their bladders before participating. Additionally, volunteers refrained from physical exercise and alcohol consumption in the 12 hours prior to the measurements. Data collection was performed using a 50 kHz frequency and disposable pre-gelled Ag/AgCl electrodes (3M Red Dot 2560, 3M, Ontario, Canada).

The measurements were carried out in the morning by trained personnel in an air-conditioned environment maintained at 23°C and with ambient humidity levels of 60–65%. Subjects were positioned supine on a non-conductive surface, without clothing or a pillow under their heads. Their arms were positioned 30° away from the chest, and their legs were placed at a 45° angle apart without contact. The skin was cleaned with 70% alcohol prior to electrode placement.

Injector electrodes were applied medial to the dorsal surfaces of the hands and feet, near the third metacarpal and metatarsophalangeal joints. Detector electrodes were positioned at the distal epiphyses of the ulna and radius at the level of the pisiform eminence, as well as midway between both malleoli. The distance between the injector and detector electrodes was standardized at 5 cm.

These participants were carefully selected following strict ethical guidelines and adhering to good medical and clinical practices outlined by the Health General Law of the Ministry of Public Health of the Republic of Cuba (Number 41, dated July 13, 1983, and updated in 2010). The research received approval from the ethics committees and scientific councils of Pediatrics Hospital “Antonio María Béguez César,” and Pediatric Hospital “Juan Martinez Maceira.” Informed consent was obtained from the parents of children and adolescent participants prior to the study's initiation.

To conduct a rigorous machine learning study, several essential steps are involved, including problem formulation, feature selection, dataset quality analysis and real-world model generalization [23,24,25,26,27,28]. Utilizing a dataset composed of 283 volunteers, a cross-validation technique was applied to ensure robust model evaluation. Specifically, 95% of the dataset was allocated for training the model, while the remaining 5% was reserved for prediction purposes. This approach was designed to avoid the overfitting problem [27,28,29,30,31,32]. Firstly, 11 features were selected, followed by a down-selection for describing the individual responses before the regression training. The features compile the following characteristics: sex, age, anthropometrics characteristics such as height, weight and weight status, bioelectrical parameters impedance (Z), corrected-reactance capacitive (Xcc), physiological characteristics such as extracellular mass (ECM), total body (TBW_Kotl_), intra (ICW_Kotl_) and extracellular (ECW_Kotl_) water according to Kotler. The phase angle (*θ*), specific resistance (*I_r_*) and reactance (*I_Xc_*) indexes (defined in Section 3) were considered as responses. All these features were taken directly from the collected data (See Supplementary Data).

The MATLAB^®^ code was utilized for regression learner purposes, leveraging the capabilities of the regression toolbox, which automatically identifies and delivers the optimal model by evaluating and comparing performance metrics [28]. This streamlined process ensures the selection of the most effective predictive model based on comprehensive data analysis.

To predict *I_r_*, the features encompass anthropometric, physiological, and bioelectric parameters, including the 11 previously mentioned features, as well as *I_Xc_* and θ. Similarly, for the prediction of *I_Xc_*, the same 11 features are utilized alongside *I_Xc_* and θ. For the prediction of the phase angle (θ), both *I_r_* and *I_Xc_* are incorporated as predictive features.

Thus, each model was trained using a comprehensive set of 13 features derived from a cohort of 283 volunteers, both male and female, aged between 2 and 18 years. This diverse dataset supports the development of robust models capable of capturing the intricate relationships among the features and enhancing predictive accuracy.

For regression models the common metrics are the coefficient of determination (*R*^2^), mean square error (MSE) and its root (RMSE), and mean absolute error (MAE) defined by equations (1):

(1) $$\begin{array}{l} \;\;\;\;\;R^{2}=1-\frac{\Sigma_{i=1}^{n}{\left(y_{i}-\hat{y_{\iota }}\right)}^{2}}{\Sigma_{i=1}^{n}{\left(y_{i}-{\bar{y}}_{i}\right)}^{2}}\\ \;\;\text{MSE}=\frac{1}{n}\sum_{i=1}^{n}{\left(y_{i}-\hat{y_{\iota }}\right)}^{2}\\ \text{RMSE}=\sqrt{\text{MSE}}\\ \;\;\text{MAE}=\frac{1}{n}\sum_{i=1}^{n}|y_{i}-\hat{y_{\iota }}| \end{array}$$

where *n* represents the number of elements in the database, *y_i_* the observed value of the dependent variable, *ŷ_i_* the predicted value of the dependent variable, and *ȳ_i_* the mean value of the observations. When *R*^2^ ≅ 1 the model tends to be perfect, analogously for small MAE, MSE and RMSE values [23,24,25,26,27,28].

As previously mentioned, the regression toolbox automatically identifies and delivers the optimal model by evaluating RMSE, along with other metrics described in equations (1).

The database collected in this study is not publicly available as it is still under investigation to extract more valuable data on the behavior of bioelectrical responses in healthy and diseased individuals, but the datasets are available from the corresponding author upon reasonable request.

### Informed consent

Informed consent has been obtained from all individuals included in this study.

### Ethical approval

The research related to human use has been complied with all relevant national regulations, institutional policies and in accordance with the tenets of the Helsinki Declaration and has been approved by the authors’ institutional review board or equivalent committee.

## Results and Discussion

### The role of specific resistance, reactance indexes and phase angle for accessing the weight status

Due to the problem of WHO to find correlations between bioelectrical and anthropometric parameters, other mixed parameters are proposed in this work by combining the bioelectrical and anthropometric parameters. For instance, it is well-know that the complex impedance (Z) is described by *Z* = *R* + *iXc*, where *R* is the resistance, *Xc* the capacitive reactance and $i=\sqrt{-1}$ . The complex impedance can be written as *Z* = |*Z*|*exp*(−*iθ*) = |*Z*|(*cosθ* + *isinθ*), where $\left|Z\right|=\sqrt{R^{2}+{\mathrm{Xc}}^{2}}$ is the absolute value of Z and *θ* the phase angle. The potential of resistance, phase angle and reactance to access the health status and cellular composition is significant, and these parameters can provide valuable information for disease diagnosis, treatment, and monitoring [29,30,31]. For now on, |*Z*| is denoted by Z.

From these parameters, the TBW_Kolt_ can be derived by sex as follows [32,33]:

Male:

(2) $${\mathrm{TBW}}_{\mathrm{Kolt}}=0.58\times \left({\mathrm{Height}}^{1.62}/Z^{0.70}\right)\times \frac{1}{1.35}+0.32\times \mathrm{Weight}-3.66$$

Female:

(3) $${\mathrm{TBW}}_{\mathrm{Kolt}}=0.76\times \left({\mathrm{Height}}^{1.99}/Z^{0.58}\right)\times \frac{1}{18.91}+0.14\times \mathrm{Weight}-0.86$$

We define specific resistance and reactance index as follows:

(4) $$I_{r}={\mathrm{TBW}}_{\mathrm{Kolt}}\times \frac{R}{{\mathrm{Height}}^{2}};I_{\mathrm{Xc}}={\mathrm{TBW}}_{\mathrm{Kolt}}\times \frac{X_{C}}{{\mathrm{Height}}^{2}}$$

In equation (3), *I_r_* and *I_Xc_* are also different by sex because of the definition of *TBW_Kolt_* by equations (2) and (3). With the measured *R*, *Xc*, Z, Weight and Height, the *TBW_Kolt_*, including *I_r_* and *I_Xc_* are derived.

In previous studies, R/Height and Xc/Height are used for analyzing the hydration status of individuals through tolerance or confidence ellipses. Both electrical magnitudes are normalized by the height of the individuals to standardize the data [1,2,29,30,31]. The electrical resistance of tissues depends on their ability to conduct electrical current. It is well known that tissues and cells with high water and electrolyte content, have low resistance, while low water areas have high resistance [1,2,29,30,31]. The capacitive reactance of tissues and cells is due to the ability of cell membranes to store and release electrical charge [1,2,29,30,31]. The cell membrane acts as a capacitor, and its capacitive reactance depends on the frequency and capacitance of the membrane [1,2,29,30,31].

To elaborate further, the use of R/Height and Xc/Height in hydration analysis allows for a more precise understanding of the body’s water distribution [1,2,29,30,31]. By normalizing these electrical properties by height, researchers can compare data across individuals of different sizes more accurately [1–2]. The low resistance indicates efficient electrical conduction, which is crucial for maintaining proper physiological functions. On the other hand, the high resistance highlights their lower water content and different conductive properties. The capacitive reactance, influenced by the cell membrane’s ability to act as a capacitor, provides insights into cellular health and integrity. This parameter is particularly important in understanding how cells interact with their environment and maintain homeostasis [1,2,30,31,32,33]. By examining *I_r_* and *I_Xc_*, one can gain a comprehensive view of both extracellular and intracellular hydration status, leading to better assessments of overall health and hydration levels.

In this section, the regression models were developed to predict the values of *I_r_*, *I_Xc_* and θ and determine their effectiveness in evaluating weight status.

**Fig.1:**
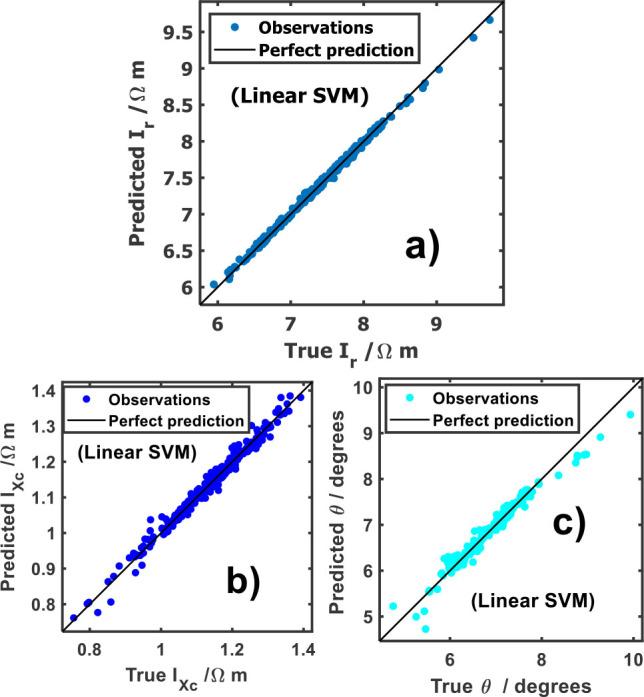
Predicted vs observable plot of: a) specific resistance index (*I_r_*), b) specific reactance index (*I_Xc_*) and c) phase angle (θ).

Figure 1a shows the predicted and true responses for the entire cohort, and the results indicate that the selected models accurately replicated the observations. As it is indicated in Figure 1, the linear support vector machine (linear SVM) model is the best approximation predicting *I_r_*, *I_Xc_* and θ. In all cases, the predicted versus true response values lie between the control line (perfect prediction), which disclose the accuracy of the lineal support vector machine learner for predicting these characteristics.

Table 1 collects the metrics of each response. The analysis of the Linear SVM regression models indicates strong predictive performance based on key evaluation metrics. The RMSE suggests that the model's predictions closely align with actual response values, minimizing significant deviations. Additionally, the high R^2^ values demonstrates that the model effectively explains the variability in the dataset. The MAE remains relatively low, although slightly higher for *I_Xc_*, indicating minor differences between predicted and actual values. These findings demonstrate that the identified responses (*I_r_*, *I_Xc_* and θ) are highly relevant and generalize well.

**Table 1:** Accuracy parameters of specific resistance-reactance indexes (*I_r_*, *I_Xc_*) and phase angle (θ) responses.

**Response**	**Model**	**RMSE**	**R^2^**	**MSE**	**MAE**
*I_r_*(Ωm)	Linear SVM	0.0320 (Ωm)	1.00	0.0010 (Ωm)	0.0240 (Ωm)
*I_Xc_* (Ωm)	Linear SVM	0.0150 (Ωm)	0.98	0.0002 (Ω^2^m^2^)	0.1064 (Ωm)
θ (º)	Linear SVM	0.0973 (º)	0.98	0.0095 (°^2^)	0.0609 (º)

**Fig.2:**
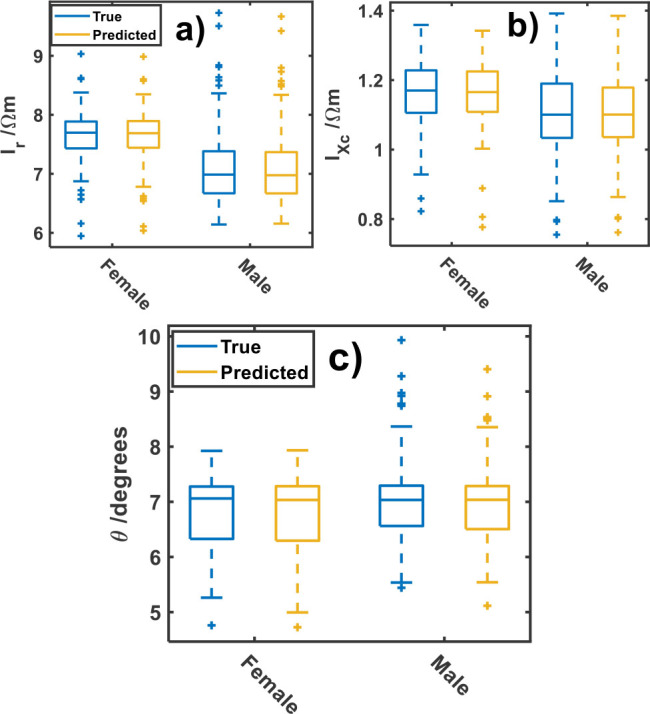
True and predicted responses versus sex of a) specific resistance index (*I_r_*), b) specific reactance index (*I_Xc_*) and c) phase angle (θ).

Figure 2 displays the dependence of specific resistance-reactance indexes and phase angle upon sex. From Figure 2a-c one can appreciate that the median values of *I_r_*, *I_Xc_* and θ for female are larger than for male volunteers.

For instance, the *I_r_* median value amounts to 7.70 ± 0.32 Ωm and 6.99 ± 0.41 Ωm (Figure 2a), for *I_Xc_* amounts to 1.17 ± 0.52 Ωm and 1.10 ± 0.63 Ωm (Figure 2b) and for phase angle 7.06 ± 0.72º, 7.03 ± 0.45º (Figure 2c) for female and male individuals, respectively. In addition, the difference between the observable and predicted values is insignificant, which points out the accuracy of the model for further predictions.

**Fig.3:**
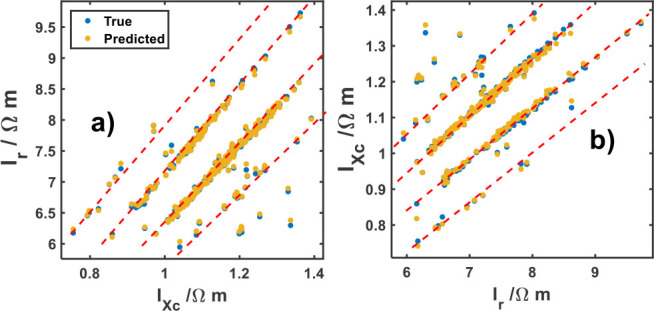
True and predicted: a) *I_r_* versus *I_Xc_* and b) *I_Xc_* versus *I_r_* bioelectrical parameters. Red lines represent a tendency between these parameters.

Figure 3 displays the relation of *I_r_* against *I_Xc_* and vice versa of their computed by equation (3) and predicted values. Note that the majority of the predicted and true data can be collected around four straight lines (dash-red lines in the Figure). There are some individuals lying between these lines; it can be explained by the fact that some individuals in the moment of the impedance measurements may be on a transition status or suffer other kinds of diseases such as malnutrition and dehydration, among others [23,24,31]. From these four straight lines in Figure 3, one can infer an association with bioimpedance vector analysis (BIVA). BIVA emphasizes the position of the impedance vector, derived from R and Xc values normalized by body height, on the R/Xc graph in relation to tolerance ellipses generated from sex, on a healthy reference population [34]. Individual and group vectors in different regions of the reference ellipses have specific body composition interpretations [35]. Further investigation will be carried out in this direction.

**Fig.4:**
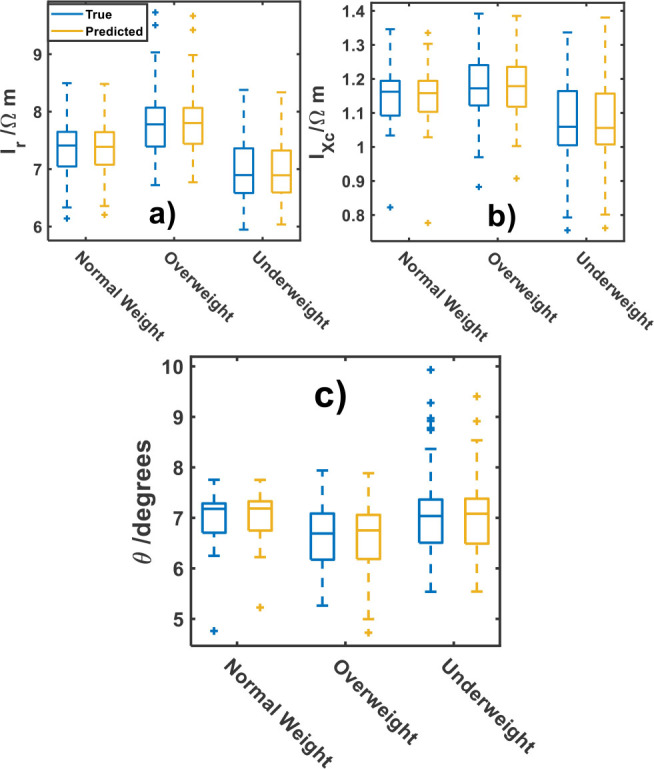
Response bioelectrical parameters of: a) *I_r_* b) *I_Xc_* and c) phase angle (θ) by weight status classes.

To complete this study, an analysis of the weight status against the specific resistance and reactance indexes, including the phase angle is developed. Figure 4 shows the trend of response parameters in relation to the weight status. As it is shown in Figure 4a and b, along the cohort, underweight status have the lowest median *I_r_* and *I_Xc_* values (6.89 ± 0.72 Ωm and 1.06 ± 0.51 Ωm, respectively); while the overweight have the highest *I_r_* and *I_Xc_* (7.78 ± 0.72 Ωm and 1.17 ± 0.51 Ωm, respectively). For individuals with normal weight, the parameters fall between those of overweight and underweight statuses. From Figure 4c it can be seen that the phase angle also has differences between weight statuses. These findings support the assertion that underweight individuals typically exhibit a larger phase angle due to the direct proportionality between Xc and the phase angle, whereas overweight individuals tend to have a lower phase angle associated with body fluid imbalances [36]. The mean values of the phase angle are 7.18 ± 0.45º, 7.04 ± 0.72º and 6.69 ± 0.58º for normal, underweight and overweight status, respectively. Furthermore, the observable and predicted parameter values closely align, validating the accuracy of the model.

Phase angle behavior throughout the lifespan has been documented [37]. In both sexes, phase angle values exhibit a pattern of progressive increase from early childhood until they remain relatively constant between the ages of 19 and 48. After 48 years of age, phase angle gradually decreases [37]. These findings hold significant relevance for clinical practice and research. Additionally, considering different age groups and sexes is essential for accurate interpretations and personalized medicine based on phase angle measurements. Due to the increment of the phase angle during the growing of healthy individuals, this parameter cannot be used as a robust predictor for accessing the health status in infant-juvenile cohorts; while the two new *I_r_* and *I_Xc_* are more robust for prognosis of weight status and diseases related in children and young individuals. Overall, the predictive models effectively replicate the observed parameters in the studied cohort.

Bearing in mind the relevance of the new parameters *I_r_* and *I_Xc_*, a systematic study to disclose the evolution of these parameters is advisable, not only in infant juvenile but also in adult-older cohorts. In addition, the relation between *I_r_* and *I_Xc_* can be used to perform studies based on tolerance ellipsoids derived from BIVA approach.

## Conclusion

In this study, a predictive regression learner model, specifically the linear support vector machine, was employed to investigate the correlation between weight status and two novel bioparameters, namely specific resistance and reactance, in an infant-juvenile cohort from the eastern Cuban region. Using various characteristics, including bioimpedance measurements, the regression learner model was trained for prediction of the phase angle, specific resistance and reactance of the volunteers with high accuracy.

The results of this study demonstrate that the variation of the aforementioned characteristics with weight status and sex across the cohort is consistent with previous literature. Additionally, two robust bioparameters derived from bioimpedance measurements and anthropometric physiological parameters were identified for predicting weight status.

The predictive models developed in this study are crucial for accurately assessing weight status and disease risks in infant and juvenile individuals residing in the eastern Cuban region. These findings provide valuable insights into the potential applications of bioimpedance measurements and bioparameters in assessing health and disease risks in this population. Overall, this study contributes to the growing body of literature on the use of bioimpedance measurements and bioparameters in predicting weight status and identifying individuals at risk of various health conditions.
